# A novel *IGHMBP2* variant and clinical diversity in Vietnamese SMARD1 and CMT2S patients

**DOI:** 10.3389/fped.2024.1165492

**Published:** 2024-02-13

**Authors:** Van Khanh Tran, My Ha Cao, Thi Thanh Hai Nguyen, Phuong Thi Le, Hai Anh Tran, Dung Chi Vu, Ha Thu Nguyen, Mai Thi Phương Nguyen, The-Hung Bui, Thanh Binh Nguyen, Thanh Van Ta, Thinh Huy Tran

**Affiliations:** ^1^ Center for Gene and Protein Research, Hanoi Medical University, Hanoi, Vietnam; ^2^ Department of Molecular Pathology, Faculty of Medical Laboratory Technology, Hanoi Medical University, Hanoi, Vietnam; ^3^ Department of Biochemistry, Hanoi Medical University, Hanoi, Vietnam; ^4^ Department of Endocrinology, Metabolism and Genetics, National Hospital of Pediatrics, Hanoi, Vietnam; ^5^ Department of Molecular Biology, National Hospital of Pediatrics, Hanoi, Vietnam; ^6^ Center for Molecular Medicine, Clinical Genetics Unit, Karolinska Institute, Karolinska University Hospital, Stockholm, Sweden; ^7^ Clinical Laboratory, Hanoi Medical University Hospital, Hanoi Medical University, Hanoi, Vietnam

**Keywords:** *IGHMBP2*, neuromuscular disorders, SMARD1, CMT2S, Charcot–Marie–Tooth type 2S, spinal muscular atrophy with respiratory distress type 1

## Abstract

**Background:**

Pathogenic variants in the *IGHMBP2* gene are associated with two distinct autosomal recessive neuromuscular disorders: spinal muscular atrophy with respiratory distress type 1 (SMARD1; OMIM #604320) and Charcot–Marie–Tooth type 2S (CMT2S; OMIM #616155). SMARD1 is a severe and fatal condition characterized by infantile-onset respiratory distress, diaphragmatic palsy, and distal muscular weakness, while CMT2S follows a milder clinical course, with slowly progressive distal muscle weakness and sensory loss, without manifestations of respiratory disorder.

**Methods:**

Whole-exome sequencing of the *IGHMBP2* gene was performed for eight Vietnamese patients with *IGHMBP2*-related neuromuscular disorders including five patients with SMARD1 and the others with CMT2S.

**Results:**

We identified one novel *IGHMBP2* variant c.1574T > C (p.Leu525Pro) in a SMARD1 patient. Besides that, two patients shared the same pathogenic variants (c.1235 + 3A > G/c.1334A > C) but presented completely different clinical courses: one with SMARD1 who deceased at 8 months of age, the other with CMT2S was alive at 3 years old without any respiratory distress.

**Conclusion:**

This study is the first to report *IGHMBP-2*-related neuromuscular disorders in Vietnam. A novel *IGHMBP2* variant c.1574T > C (p.Leu525Pro) expressing SMARD1 phenotype was detected. The presence of three patients with the same genotype but distinct clinical outcomes suggested the interaction of variants and other factors including relating modified genes in the mechanism of various phenotypes.

## 1 Introduction

Spinal muscular atrophy with respiratory distress type 1 (SMARD1, OMIM#604320) is a neuromuscular disorder resulting from the degeneration of alpha motor neurons in the anterior horn cells of the spinal cord in which severely impaired helicase and ATPase activities are the primary pathological mechanism of SMARD1 (1). Critical clinical presentations include (1) manifestation age of respiratory distress between 6 weeks and 6 months, (2) presence of diaphragmatic paralysis, and (3) distal muscular weakness or (4) intrauterine growth retardation (2, 3); most SMARD1 patients died within the first few years of life. Charcot–Marie–Tooth type 2S (CMT2S, OMIM#616155) is an axonal neuropathy in juvenile or adolescent onset with slowly progressive distal muscle weakness and sensory loss (4). CMT2S generally exhibits less severe symptoms than SMARD1 with no respiratory impairment. The prevalence of both disorders is scarce and has yet to be determined.

SMARD1 and CMT2S are known to be associated with homozygous or compound heterozygous pathogenic variants in *IGHMBP2*. Immunoglobulin mu-binding protein 2 (IGHMBP2), encoded by *IGHMBP2* in chromosome 11, is a ubiquitously expressed helicase in the human body, with the highest expression in the cerebellum (4), which functions to unwind both DNA and RNA duplexes (5). The exact mechanism in which this protein affects the motor neuron function is unknown; however, Grohmann et al. (6) stated that IGHMBP2 and SMN share an important role in motor neuron maintenance in mammals.

In this study, we describe eight patients presented with *IGHMBP2*-related neuromuscular disorders. Six patients presented with muscle weakness of both limbs and infancy-onset respiratory distress, while the others exhibited lower limb muscle atrophy. Thorough clinical characterization was conducted and discussed further together with genotyping using whole-exome sequencing (WES) for definitive diagnosis. The clinical report highlights the importance of molecular genetic testing in diagnosing rare hereditable diseases as well as thorough genetic counseling and prenatal diagnosis for the affected family.

## 2 Materials and methods

### 2.1 Patients collection

Of the 35 unrelated patients meeting SMARD1 or CMT2S criteria, the whole-exome sequencing screening was performed to identify *IGHMBP2* variants. Eight patients of seven families including six patients (two patients in the same family) with SMARD1 and two patients with CMT2S were defined. These families with *IGHMBP2*-related neuromuscular disorders were recruited for target sequencing of the *IGHMBP2* gene, which was defined from the result of the patient's whole-exome sequencing. All patients and their families were counseled to provide informed consent to participate in the study as required by the Vietnam Medical Ethics Council.

### 2.2 Genetic techniques

Samples of 2 ml peripheral blood with EDTA anticoagulation were obtained from the patients and their family members. The genomic DNA of participants was extracted from peripheral blood using QIAGEN Puregene Core Kit C (Qiagen, Valencia, CA, USA) according to the manufacturer's protocol. The concentration of DNA was then determined by a Thermo Scientific NanoDrop spectrophotometer (Waltham, MA, USA).

Patients’ DNA samples were whole-exome sequenced by using the Agilent SureSelect Target Enrichment kit (Illumina, CA, USA) for the library constructions and the SureSelect V7-Post kit (Illumina, CA, USA) for sequencing. The techniques were performed on the Illumina sequencing machine (Illumina, CA, USA). Targeted panel sequencing was employed for *IGHMBP2* analysis of the patients, and the initial result returned the suspected disease-causing variants.

Sanger direct sequencing was performed on an ABI PRISM 3500 Genetic Analyzer machine (Thermo Fisher Scientific Inc., USA) for confirmation of patients’ WES results and genetic analysis of family members.

### 2.3 Data management and variant analysis

WES data were analyzed by BWA version 0.7.12 (http://bio-bwa.sourceforge.net/bwa.shtml), Picard version 1.130 (http://broadinstitute.github.io/picard/), GATK version 3.4.0 (https://www.broadinstitute.org/gatk/), and SnpEff version 4.1g (http://snpeff.sourceforge.net/SnpEff.html) software for *IGHMBP2* genetic variants and their potential effects. The Sanger sequencing data were analyzed by using BioEdit 7.2.5 software.

To assess the pathogenicity and novelty of the *IGHMBP2* variants, we used the Human Gene Mutation Database, Leiden open variants database (LOVD), and ClinVar as reference databases and followed instructions from the 2015 American College of Medical Genetics Guidelines (7).

## 3 Results

### 3.1 Clinical presentations

Of the 35 unrelated patients meeting SMARD1 or CMT2S criteria, the whole-exome sequencing screening identified homozygous or compound heterozygous *IGHMBP2* variants in eight patients of seven families including six patients (of which two were in the same family) with SMARD1 and two patients with CMT2S. Major clinical features and genetic findings are summarized in Table 1.

**Table 1:** Clinical characteristics and *IGHMBP2* variants found in eight patients.

	Patient 1	Patient 2	Patient 3	Patient 4	Patient 5	Patient 6	Patient 7	Patient 8
Sex/age	F/4	F/deceased	F/deceased	M/13	M/3	M/deceased at 8 months	F/deceased at 7 months	F/deceased at 2 years
*IGHMBP2* variants	c.1334A > C/c.1334A > C	c.1235 + 3A > G/c.1574T > C*	c.1235 + 3A > G/c.1334A > C	c.1235 + 3A > G/c.2623C > T	c.1235 + 3A > G/c.1334A > C	c.1813C > T/c.1813C > T	c.1813C > T/c.1813C > T	c.1235 + 3A > G/c.1334A > C
Protein	p.His445Pro	Splicing/p.Leu525Pro	Splicing/p.His445Pro	Splicing/p.Arg788Ter	Splicing/p.His445Pro	p.Arg605Ter	p.Arg605Ter	p.His445Pro
Prenatal signs	Intrauterine growth delay	No	No	No	No	Fetal growth retardation after 20 weeks	Fetal growth retardation after 20 weeks	
Weight at birth	2,300 g	3,200 g	3,200 g	3,300 g	3,700 g	1,900 g		
Apgar score	10	7 (weak cry)	10	10	10	10	10	
Onset of first symptoms	4 months, cyanosis and dyspnea when crying	3 months, short breath, muscle weakness	5 months, cannot move without support, dyspnea	2 years, leg muscle weakness	4 months, leg muscle weakness	From birth, leg muscle weakness	From birth, leg muscle weakness	2.5 months, dyspnea; 3 months, leg muscle weakness; 5 months, arm muscle weakness
Failure to thrive	Yes, 4 months	Yes, 7 months	Yes, 5 months	No	No	Yes	Yes	
Respiratory distress	Yes, 4 months	Yes, 3 months	Yes, 5 months	No	No	Yes, 7 months	Yes, 7 months	
Mechanical ventilation	Tracheostomy 15 months	Tracheostomy 8 months	Mask ventilation	No	No	Tracheostomy 7 months	Tracheostomy 7 months	
Muscle weakness onset	10 months, upper and lower limbs	3 months, lower limbs	5 months, upper and lower limbs	2 years, lower limbs (distal then proximal)	4 months, upper and lower limbs	From birth, lower limbs	From birth, lower limbs	2.5 months, respiratory muscle
Deep tendon reflexes	Absent	/	/	Markedly decreased	Absent			
Pain sensation	Markedly decreased	/	/	Decreased	Decreased			
Holding head upright while sitting	No	No	No	Yes, around 2 years	Yes			Yes
Sitting without support	No	No	No	Yes, around 2 years	Need help to transit from lying to sitting			
Musculoskeletal deformities	No	No	No	No	Suspected scoliosis			
Reduce facial expression	No	No	No	No	No			
Autonomic nervous system dysfunction	No	No	No	No	No			
Current status	Alive, 4 years, mechanical ventilation, motionless	Deceased at 9 months	Deceased at 8 months	Alive, 13 years, wheelchair-bound, normal cognitive function	Alive, 3 years, normal cognitive function, fluent speaking ability.	Deceased at 8 months	Deceased at 7 months	Deceased at 2 years

/, no record.

* For novel variant.

#### 3.1.1 Patient 1

**Figure 1:**
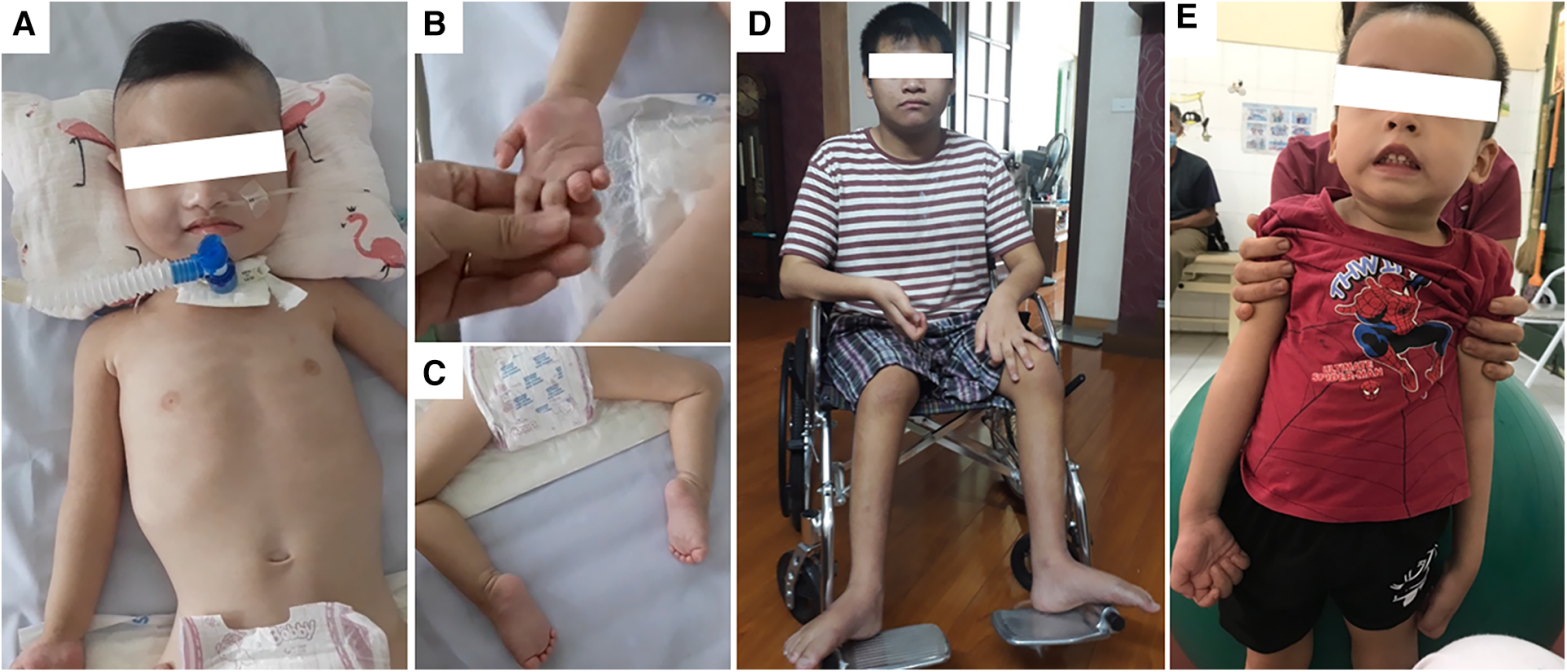
Photographs of patient 1 (**A–C**); patient 4 (**D**), and patient 5 (**E**). (**A**) Patient 1 (4 years old) was fully dependent on mechanical ventilation; (**B**) fatty pads of the fingers; (**C**) talipes equinovarus as the consequence of lower limbs muscle weakness. (**D**) Patient 4 (13 years old) had been wheelchair-bound since the age of 9. (**E**) Patient 5 (3 years old) had muscle weakness in both arms and legs and required assistance to stand up as shown in the image.

The female patient was born to a full-term pregnancy of healthy non-consanguineous Vietnamese parents. Intrauterine growth restriction was noted, and the resulting birth weight was 2,300 g. At 4 months of age, she was noticed to experience dyspnea and cyanosis when crying; she was hospitalized immediately with the diagnosis of pneumonia. The condition was exacerbated gradually to respiratory distress; mechanical ventilation was required to assist her breathing. During the first year of hospitalization, two attempts at weaning her from mechanical ventilation were unsuccessful; thus, at 15 months of age, a tracheostomy was performed. The patient's muscle weakness became noticeable from the age of 10 months as she could not lift either her head or limbs by herself. At the time of the study, the patient was 4 years old; clinical examination revealed muscle atrophy of arms and legs with hardened joints, paralysis of four limbs, fatty pads of the fingers, and talipes equinovarus (Figures 1A–C and Table 1).

#### 3.1.2 Patient 2

The patient was the first child of unrelated Vietnamese parents following an uneventful pregnancy. Her birth weight and length were 3,200 g and 50 cm**,** respectively. She had a weak cry and slightly decreased muscular tone in both legs from birth. At 3 months of age, hypotonia of both legs was observed, which gradually became atrophic as she could not sit or move her legs without support. The patient was also noted for breathing difficulty when swallowing and crying and was hospitalized at 4 months of age. She displayed no abnormal dysfunction of the autonomic nervous system. At 8 months of age, a tracheostomy was performed when she exhibited severe respiratory distress. After 3 weeks of extensive mechanical ventilation and medical care, the patient expired at 9 months of age (Table 1).

#### 3.1.3 Patient 3

Patient 3 was born after full-term pregnancy; parents denied any consanguinity. She presented with normal growth in the first 3 months. However, at 5 months of age, she exhibited insufficient weight gain and delayed motor development (e.g., inability to lift her head or flip her body without support). When admitted to the hospital, clinical examination revealed hypotonia of both arms and legs. Wheezing and dyspnea during crying were occasionally observed but she only needed an oxygen mask to elevate her breathing. However, at 8 months of age, her respiratory symptoms and muscle weakness worsened and she died of acute respiratory distress (Table 1).

#### 3.1.4 Patient 4

Patient 4 was the second child of non-consanguineous Vietnamese parents. Since birth, he grew up normally with no signs of muscle weakness. However, the motor development milestone was delayed as he was only able to walk from 2 years of age. Toe walking was also noted. Since his walking capacity decreased progressively, he was appointed for regular check-ups and required a walking frame for ambulation since 9 years of age. The patient was 13 years old and wheelchair-bound (Figure 1D) at the time of this study. He had normal cognitive function and no muscle weakness in both arms. There were no notable respiratory disorders in this patient (Table 1).

#### 3.1.5 Patient 5

Patient 5 was also born after a full-term pregnancy with neither abnormal prenatal signs nor failure to thrive. The first symptom appeared at 4 months old with mild muscle weakness in both legs; however, the child was still able to hold his head upright and turn over by himself. Muscle weakness of the legs gradually became more apparent and at the age of 1, and arm muscle tone loss was also observed. At the time of the research, the patient was 3 years old with normal cognitive function and an inability to raise arms or legs, resulting in walking with constant support (Figure 1E). Clinical examination revealed that when the patient was assisted from lying to sitting, his sitting posture suggested possible scoliosis. No respiratory disorder was witnessed (Table 1).

#### 3.1.6 Patient 6

Patient 6 was an 8-month-old male who was the first child of healthy and unrelated parents. Intrauterine growth restriction was noted from 20 weeks gestation, and he was born at 36 weeks of gestation with a birth weight of 1,900 g and dyspnea. He was hospitalized for 14 days after birth. At the age of 8 months, the patient was admitted to the hospital owing to respiratory distress and was diagnosed with pneumonia. He could not lift his limbs and had growth retardation. He was subsequently intubated and mechanical ventilation was initiated. After 2 weeks of extensive mechanical ventilation and medical care, the patient expired at 8 months of age.

#### 3.1.7 Patient 7

Patient 7 was a 7-month-old female who was the second child of healthy and unrelated parents and was the younger sister of patient 6 with the same symptoms. Intrauterine growth restriction was noted from 20 weeks gestation, and she was born with a birth lightweight and dyspnea. At the age of 7 months, she had difficulty to suckle and swallow, shortness of breath, wheezing, pale skin, and muscle weakness. Then, she was admitted to the hospital owing to respiratory distress and was diagnosed with pneumonia and myasthenia gravis. She was subsequently intubated and mechanical ventilation was initiated. After 2 weeks of extensive mechanical ventilation and medical care, the patient expired at 7 months of age.

#### 3.1.8 Patient 8

Patient 8 was also born after a full-term pregnancy with neither abnormal prenatal signs nor failure to thrive. The first symptom appeared at 2.5 months old with dyspnea and respiratory distress, and mild muscle weakness of both legs appeared at 3 months of age. Muscle weakness in arms muscle was also observed at 5 months old; however, the child was still able to hold her head upright and turn over by himself. The patient deceased at 2 years of age.

### 3.2 Genetic analysis and genetic counseling

**Figure 2:**
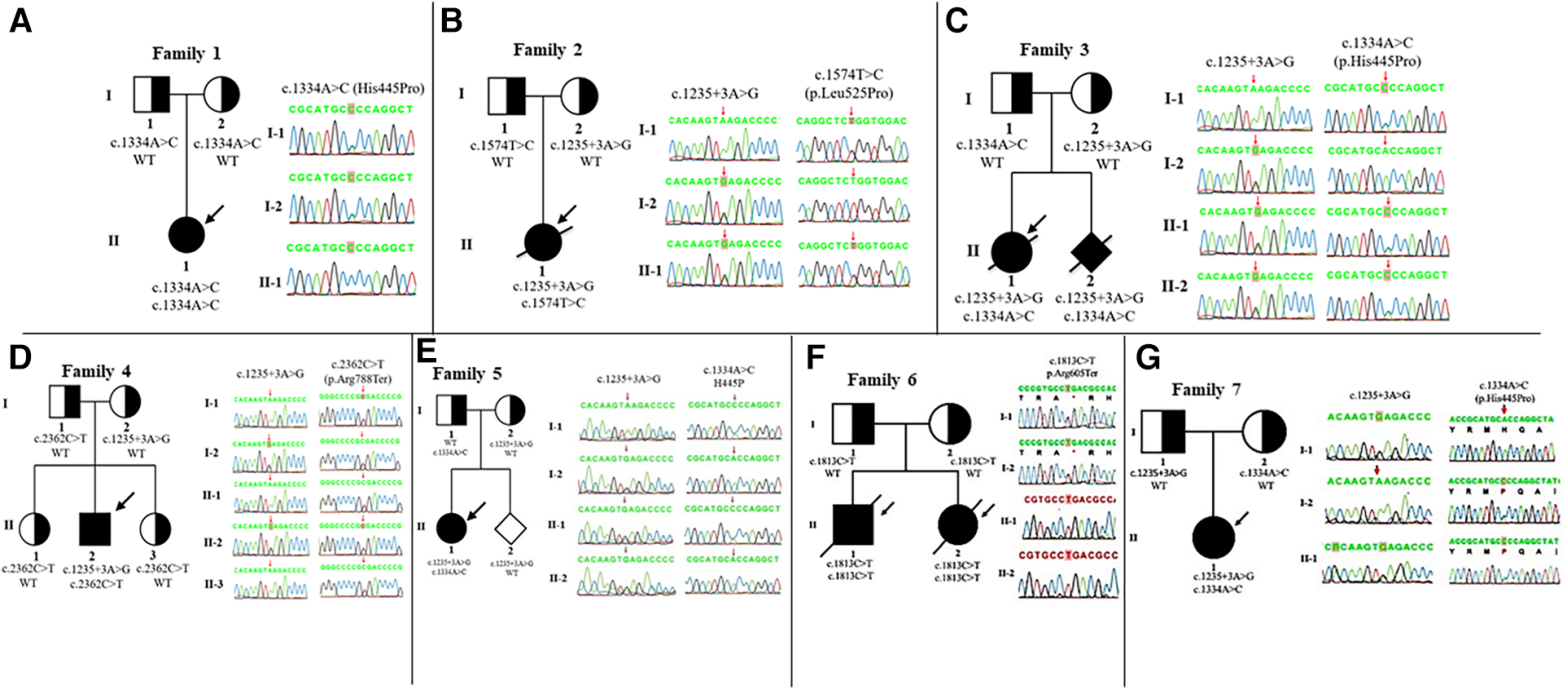
*IGHMBP2* analysis and pedigree of the families. Clear, half-shaded, and shaded symbols present unaffected, carrier, and affected individuals, respectively. Variants of family 1 (A), 2 (B), 3 (C), 4 (D), 5 (E), 6 (F), and 7 (G) are inherited from their parents, respectively. The arrows point to the probands.

Whole-exome sequencing was performed on eight patients and five *IGHMBP2* variants were identified: c.1235 + 3A > G, c.1334A > C (p.His445Pro), c.1574T > C (p.Leu525Pro), c.1813C > T (p.Arg605Ter), and c.2362C > T (p.Arg788Ter) (Table 1). Patients 1, 6, and 7 were homozygous for an *IGHMBP2* variant, while the others had compound heterozygous variants. Especially, we detected novel heterozygous variants c.1574T > C (p.Leu525Pro) in patient 2 with SMARD1 exhibition. Notably, patients 3 and 8 (with SMARD1 diagnosis) and patient 5 (with CMT2S diagnosis) shared the same *IGHMBP2* variants [c.1235 + 3A > G, c.1334A > C (p.His445Pro)]. Sanger sequencing was performed for confirmation of each patient and genetic analysis of family members. The result showed that all parents were carriers. Detailed clinical manifestations and genetic analysis are shown in Table 1, Figure 2, and Supplementary Table S1.

## 4 Discussion

Charcot–Marie–Tooth (CMT) is also known as hereditary motor sensory neuropathy (HMSN). The diagnostic criteria of CMT2S consisted of normal nerve conduction study velocity and reduced amplitudes of the motor and sensory potentials (8). SMARD1 belonged to the group of dSMA (distal muscular atrophy), also called dHMN (distal hereditary motor neuropathy). The diagnostic criteria of SMARD1 have evolved from only clinical evaluations (9) to including pathological and electromyography criteria (10). Theoretically, dHMN is in contrast with CMT and other hereditary sensory neuropathies where sensory involvement forms a significant component of the disease (11). Nonetheless, clinically, dHMN can hardly be distinguished from CMT neuropathy because clinical examination could miss sensory symptoms, thereby misdiagnosing CMT as dHMN; also patients with dHMN can develop sensory symptoms later in life and then be classified to be CMT. The clinical course of eight patients, including five heterozygosities and three homozygosities, showed that six patients (all homozygosities—patients 1, 6, and 7; three heterozygosities—patients 2, 3, and 8) had significant hypotonia before 1 year of age; they also suffered from respiratory distress, which was the cause of death in five of the six patients. This presentation complies with clinical criteria provided by Eckart et al. (2), suggesting that these six patients had SMARD1 that has not been reported previously in Vietnam. Cottenie et al. (4) recorded that in patients having CMT2S with *IGHMBP2* variants, the age of onset varied from 1 to sooner than 10 years old; all patients exhibited significant muscle weakness and absent reflexes but no respiratory problems. Similar clinical presentations found in patients 4 and 5 allowed us to conclude the diagnosis of CMT2S. In our study, no patients had reduced facial expression or dysfunctional autonomous systems.

The *IGHMBP2* gene contains 15 exons and encodes a protein of 993 amino acids (6, 12). The product of the *IGHMBP2* gene is the DNA/RNA helicase of the SF1 superfamily that is involved in the regulation of pre-mRNA processing and transcription (13). This enzyme includes a putative DNA helicase domain (amino acids 19–641) containing a DEAD/H box helicase and an AAA ATPase DEXDc region, a single-stranded nucleic acid-binding R3H motif (amino acids 726–784), and an AN1-like zinc-finger region (amino acid 897–940) (14, 15). In previous studies of patients with SMARD1 or CMT2S, mutations of IGHMBP2 have been found predominantly located in the helicase domain. In this study, targeted panel sequencing was employed for *IGHMBP2* analysis of the patients, and the initial result returned five suspected disease-causing variants. To assess the pathogenicity and novelty of the *IGHMBP2* variants, we used the Human Gene Mutation Database, LOVD, and ClinVar as reference databases and followed instructions from the 2015 American College of Medical Genetics Guidelines (7). The novel variant c.1574T > C (p.Leu525Pro) was found in patient 2 in compound heterozygosity with a common variant (c.1235 + 3A > G, aberrant splicing). The patient presented with severe respiratory distress symptoms as soon as giving birth and deceased at 9 months of age. The variant is located in the helicase domain, which is the same common residue as the previously discovered variants and was classified as “likely pathogenic” (PM1, PM2, PM3, PP3).

The c.1235 + 3A > G variant appeared with the highest frequency (five out of eight patients) in this study and has previously been linked to CMT2S but exhibited SMARD1 phenotype in compound heterozygosity with other variants including c.1574T > C (p.Leu525Pro), c.1334A > C (p.His445Pro), or exhibited CMT2S phenotype in combination with c.2623C > T (p.Arg788Ter) variant. Interestingly, genetics analysis revealed that patients 3, 5, and 8 shared compound heterozygous variants [c.1235 + 3A > G, c.1334A > C (p.His445Pro)], but the clinical presentation of patients 3 and 8 suggested SMARD1, and patient 5 was diagnosed with CMT2S. In addition, the variant c.1813C > T found in the homozygous state of patients 6 and 7 exhibited the SMARD1 phenotype in contrast to previous study (4) that showed CMT2S manifestation. These observations are in line with many previous studies revealing that patients with SMARD1 or CMT2S are not distinguishable at the genetic level, whereas a correlation between mutations at the protein level and clinical severity remains unclear (4, 16, 17).

The mechanism underlying the two distinct phenotypes was studied by Cottenie et al. (4) who first identified CMT2S. Different residual enzymatic activity of IGHMBP2 might be the cause for the heterogeneity in clinical outcomes. Previous studies showed that *IGHMBP2* mRNA levels were preserved in SMARD1/CMT2S patients despite the reduction of protein levels in both phenotypes compared with healthy individuals, and in SMARD1 subjects, the protein level was lower than that in CMT2S patients (3, 4, 18). Thus, the reduction in IGHMBP2 protein might be part of the pathogenesis of SMARD1/CMT2S that could result from post-translational degradation (4) or inhibition of nonsense-mediated RNA decay (NMD) (18). However, these studies have not elucidated why patients with the same variants would have distinct clinical diagnoses. Therefore, we hypothesized that there might be the role of modifier genes that resulted in variable expressivity. It is well-studied that the idea of monogenic diseases was not quite accurate; there might be the interactions of modifier genes and other genetic backgrounds that resulted in the disease (19, 20). Much research has sketched out the picture of how a phenotype can vary due to gene interactions, epigenetics, and stochasticity for the same pathogenic variants in a single gene, thereby leading to the presence of incomplete penetrance and variable expressivity (21, 22). Especially, the interaction of other proteins involved in the regulation of pre-mRNA processing and transcription like IGHMBP2 protein may distribute to clinical diversity such as GARS (glycyl-tRNA synthetase), AARS (aminoacyl-tRNA synthetases), HSPB1 (heat shock 27 kDa protein 1), and HSPB8 (heat shock 22 kDa protein 8). All of these were linked to both axonal CMT and dHMN phenotype (23–28). Dysfunction of RNA processing pathways of gene interaction may affect the peripheral nerve and central nervous system and accordingly contribute to phenotypic variability. Further studies should investigate to identify factors contributing to this various susceptibility such as mutant analysis in other related genes or regulatory region of the *IGHMBP2* gene, expression of mRNA, and protein level of the proteins that may explore the interaction between genotype and phenotype. However, since the clinical diagnostic criteria of SMARD1 and CMT2S are partly parallel, and there might be modifier genes affecting the phenotype expression, we suggested that SMARD1 and CMT2S can be of a phenotypic spectrum instead of two completely different diagnoses. This concept could be implausible at first, but it deserves consideration in further in-depth analysis.

## 5 Conclusion

In this study, we present an original report on *IGHMBP-2*-related neuromuscular disorders in Vietnam. In our cohort, a novel recessive IGHMBP2 variant was detected in the compound heterozygosity of a SMARD1 patient. Given the significant clinical diversity observed, the identification of potential genetic factors is necessary to determine patient phenotypes. Moreover, further investigation is required to explore the contributory factors for genetic testing and inheritance counseling, as well as potentially therapeutic methods to improve patient quality of life.

## Data Availability

The datasets presented in this article are not readily available because of ethical and privacy restrictions. Requests to access the datasets should be directed to the corresponding author.
