# Supplementary material for: A novel IGHMBP2 variant and clinical diversity in Vietnamese SMARD1 and CMT2S patients
Source: Front Pediatr. 2024 Feb 13;12:1165492. doi: 10.3389/fped.2024.1165492 (PMC10896978; doi:10.3389/fped.2024.1165492)
Supplement: Supplementary file 1 [file Table1.docx]

| **Variants** | **Location** | **Amino acid change** | **GnomAD**  **(South Asian)** | **Mutationtaster** | **FATHMM** | **DANN** | **In vitro evidence** | **Previously reported** |
| --- | --- | --- | --- | --- | --- | --- | --- | --- |
| c.1235+3A>G | Intron 8 | Splice site | − | − | − | 0.6139 | Yes | [3] |
| c.1334A>C | Exon 9 | p.His445Pro | − | Disease causing | Damaging | 0.9879 | − | [3] |
| c.1574T>C (novel) | Exon 11 | p.Leu525Pro | − | Disease causing | Damaging | 0.9989 | − | − |
| c.2362C>T | Exon 13 | p.Arg788Ter | − | Disease causing | Neutral | 0.9623 | Yes | [7]  [8] |
| c.1813C>T | Exon 13 | p.Arg605Ter |  |  |  |  |  | [9] |

**Supplementary Table 1**. Information of *IGHMBP2* variants found in eight patients

**Supplementary Reference**

[3] Guenther U-P, Varon R, Schlicke M, Dutrannoy V, Volk A, Hübner C, et al. Clinical and mutational profile in spinal muscular atrophy with respiratory distress (SMARD): defining novel phenotypes through hierarchical cluster analysis. Hum Mut 2007.

[7] Grohmann K, Varon R, Stolz P, Schuelke M, Janetzki C, Bertini E, et al. Infantile spinal muscular atrophy with respiratory distress type 1 (SMARD1). Ann Neurol 2003;54:719–24.

[8] Viguier A, Lauwers-Cances V, Cintas P, Manel V, Peudenier S, Desguerre I, et al. Spinal muscular atrophy with respiratory distress type 1: A multicenter retrospective study. Neuromuscul Disord 2019;29:114–26.

[9] Cottenie E, Kochanski A, Jordanova A, et al. Truncating and missense mutations in IGHMBP2 cause Charcot-Marie Tooth disease type 2. *Am J Hum Genet*. Nov 06 2014;95(5):590-601. doi:10.1016/j.ajhg.2014.10.002 <https://www.ncbi.nlm.nih.gov/pmc/articles/PMC4225647/>
